# Socio-Cultural and Environmental Factors that Influence Weight-Related Behaviors: Focus Group Results from African-American Girls and Their Mothers

**DOI:** 10.3390/ijerph15071354

**Published:** 2018-06-28

**Authors:** Daheia J. Barr-Anderson, Alexis W. Adams-Wynn, Olubunmi Orekoya, Sofiya Alhassan

**Affiliations:** 1 School of Kinesiology, University of Minnesota, Minneapolis, MN 55455, USA; 2 Detroit Health Department, Detroit, MI 48207, USA; adamsal@detroitmi.gov; 3 Metropolitan Hospital Center, New York, NY, 10029 USA; orekoyao@nychhc.org; 4 Department of Kinesiology, University of Massachusetts Amherst, Amherst, MA 01003, USA; alhassan@umass.edu

**Keywords:** obesity, physical activity, diet, culturally appropriate

## Abstract

African-American girls experience higher rates of obesity than other youth and are more likely to live in environments that may inhibit healthy lifestyles. Focus groups with African-American girls (14.2 ± 2.36 years) and their mothers were conducted to explore socio-cultural and physical factors within the home, neighborhood, and school environments that influence physical activity (PA) and food choices (i.e., availability and accessibility). Being active at home was dependent on availability of unstructured PA, possibility of activity with family/friends/pet, structured sports in the community, and perceived safety of neighborhood. Girls reported unhealthy foods and excessive snacking as issues at home while citing choice of school meals vs. vending machine items and easy accessibility to fast food restaurants as concerns at school. Learning more about the PA and food environments is a fundamental step to develop effective and innovative, environmental strategies to address unhealthy weight-related behaviors in this population.

## 1. Introduction

Obesity is a significant public health problem in the United States as it is associated with adverse consequences, such as cardiovascular, pulmonary, gastrointestinal, metabolic and neurologic diseases [1]. Since the 1980s, the rate of obesity in African-American girls has steadily increased from 16.3% to a peak prevalence of almost 30% in 2008 [2] with a current rate of approximately 25% [3]. Other racial/ethnic groups, males and females, and people across the lifespan have also experienced drastic increases in obesity. However, African-American girls have the highest likelihood of becoming obese as an adult compared to other groups with almost 55% of African-American women classified as obese [3]. Obesity in childhood is predictive of obesity in adulthood [4], which makes it pressingly clear that efforts to address excessive weight gain in African-American females are needed.

There is a need to understand the interwoven relationship between socio-cultural, behavioral, and environmental factors (i.e., related to availability and accessibility) and how these factors influence obesity rates particularly among African-American girls. Obesity in adolescents are associated with a complex interplay of factors related to physical activity (PA) and eating behaviors [5]. More studies have examined individual, behavioral, and socio-cultural influences of PA [6] and eating [7,8] relative to environmental factors. In more recent years, the influence of environmental factors on obesity has become of increasing interest [9,10,11]. Research has found physical, sociocultural, economic, and policy environmental correlates to be associated with PA [12] and eating behaviors [13]. However, it has been suggested that more research is needed to better conceptualize social and physical environments of youth and how they relate to behavior [14]. 

In general, African-Americans tend to live in racially segregated communities that are less supportive of PA and healthy eating than whites [15]. African-American girls’ PA level is more influenced by environmental factors, such as availability of home equipment, access to physical parks and gyms, and participation on sports teams, compared to white girls [16]. Despite this, most research that identifies environmental correlates of obesity have drawn these conclusions from predominantly white populations [17]. Moreover, studies have shown that African-American girls are less likely to be active compared to white girls [18,19] and boys report more tangible support for PA than girls, including parental support and constructive feedback from parents and caregivers [20,21,22]. A focus group study found that cultural beliefs influence perceptions of weight and healthy lifestyle in African-American girls [17]. With the high prevalence of obesity in African-American girls, the potential influence of environmental factors on this high prevalence, and the importance of exploring the intersection of race/ethnicity and gender on health behaviors, there is a need to study this topic in this population.

The current study was a qualitative exploration of family/home, school, and neighborhood environmental factors that may influence African-American girls’ choices related to PA and eating. Focus groups were conducted with African-American girls aged 11–18 years and their female caregivers. This study extends the current literature because multi-level environmental variables related to weight-related behavior were collectively explored in a sample of African-American adolescent girls. Findings from this study may help inform culturally-competent preventive strategies to reduce obesity and address disparities in weight-related comorbidities in the target population.

## 2. Materials and Methods

### 2.1. Participants

Ten focus groups were conducted with self-identified African-American girls aged 11–18 years (n = 34) and their female caregivers (n = 24), who identified as one of the primary caregivers of the participating girl (hereafter referred to as “mothers”). Additional inclusion criteria for both daughters and mothers were the ability to be physically active and not following a restricted diet.

Eighteen middle and 16 high school African-American girls participated in focus groups with 4–7 girls (mean = 6 girls). Due to developmental differences between younger and older adolescent girls, focus groups for middle and high school girls were held separately (three focus groups for each grade level). Mean age for girls was 14.2 ± 2.36 years (middle school = 12.2 ± 1.15 years; high school = 16.4 ± 1.02 years). Four focus groups were conducted with a total of 12 mothers of middle school girls and a total of 12 mothers of high school girls.

### 2.2. Procedure

Participants were recruited using audio, print, and electronic methods. Radio advertisements were announced on a popular, local music station with a predominantly African-American listenership. Advertisements were placed in newspapers and other print and electronic media with predominant African-American readership. Recruitment materials were mailed to community organizations (i.e., churches and businesses that serve youth or families), African-American professional groups, and alumni sororities and fraternities to share with members. Electronic strategies included publicizing on Facebook and engaging community organizations and university listservs to distribute recruitment information to subscribers.

Potential participants called the study hotline and were screened by a trained research assistant. For informed consent purposes, mothers were screened first and upon confirmation of the mother’s eligibility, the daughter’s eligibility was verified. Eligible daughter/mother pairs signed up for the appropriate focus groups. Enrolled participants received a letter reiterating the purpose of the group discussion and outlining pertinent details (i.e., date/location/time and bus route information). A reminder email was sent one week prior and a reminder phone call was made two days before the assigned focus group. If a daughter and/or mother missed her allotted focus group, every attempt was made to reassign the participant to another group discussion. All focus groups were conducted within a 4-week period by research study staff trained to conduct focus groups.

Mothers and daughters provided written informed consent and assent, respectively. University of Minnesota Institutional Review Board approved the protocol and ethical procedures for this study (approval # 0905S65182).

### 2.3. Focus Group Questions

A focus group guide was developed that explored: (1) perceptions of PA, food, and weight and their role in family, school, and neighborhood/community life (i.e., PA viewed as positive/negative and foods that are considered healthy/unhealthy); (2) family culture and rules related to PA and eating (i.e., watching TV while eating allowed/forbidden and using food as a reward); (3) neighborhood characteristics and safety; and (4) availability of PA and food resources at home, at school, and in the neighborhood. During the developmental phase of the study, focus group questions were pilot tested with a sample of adolescent girls and their mothers. Focus group questions and protocol were revised, as needed.

The focus group questions were delivered in a semi-structured format by lead author (DJBA) and an assistant moderator, who recorded notes. Each session lasted for approximately an hour. Focus groups were conducted in a church, library, or non-profit family service organization nestled in predominantly African-American communities. Two digital audio recorders were used to record all discussions. Demographic information was collected from participants at the end of each focus group.

### 2.4. Data Analysis

Transcript data were analyzed using a grounded theory approach [23,24], a common methodology for analyzing focus group data. This approach includes 5 steps of describing, organizing, connecting, corroborating, and representing the account or story/narrative. Per the focus group protocol, assistant moderators typed up handwritten notes and observations from the focus groups within 48 h. Within two weeks, these same moderators transcribed the audio files verbatim, using one audio file for transcription and the second file to review the transcripts. A co-author (A.W.A.-W.) proofread the transcripts by listening to a recording of each focus group while reading its transcript. Any discrepancies were resolved by the assistant moderator and A.W.A.-W. Once transcription was complete, D.J.B.-A. and A.W.A.-W. reviewed the transcripts and developed a codebook. Once the codebook was finished, resulting in 193 codes, the same authors independently reviewed the transcripts and assigned codes to applicable transcript text. A data dictionary was created to define each code as it relates specifically to the text. These codes were entered into NVIVO 8 software, using a compare and contrast method. Summaries were prepared for each code to determine thematic saturation by D.J.B.-A. and A.W.A.-W. during meetings to discuss the major themes. Each coder identified themes independently, compared findings, and came to mutual agreement through discussion. Member checking, a technique in which researchers improve the accuracy, credibility, and validity of qualitative data by presenting the information to the participants after data analysis, was performed. All participants who were involved in the focus groups were mailed summaries of each of the major themes and asked to verify the authenticity and accuracy of such summaries. There were zero requests to change the summaries. When presenting the results below, the term “commonly” is used when three or more participants stated the same idea.

## 3. Results

The most prevalent factors discussed by daughters and mothers were related to home and school PA environments, home and school food environments, and rules pertaining to PA, food, and sedentary behavior within the home environment.

### 3.1. Home PA Environment

Girls and mothers described four specific influences for increasing PA in their home environment: unstructured PA; family members, friends, peers and pets with whom they can be active; structured sports within the community; and perceived safety related to neighborhood crime and violence. Table 1 presents quotes that illustrate influencing factors.

### 3.2. School PA Environment

The majority of the girls identified a range of PA resources available to them at school. Schools were recognized as offering gyms, tennis courts, dance studios, basketball courts, parks, trails, and football fields. Although the space and activities were available and some of the girls reported utilizing these resources, it was not clear how often school facilities were utilized by the girls. However, mothers seem to be aware of the activities available to their daughters and encouraged participation (Table 1—School PA environment). Several girls also described barriers, such as weather, peer perception, convenience, and time constraints, that prevented them from utilizing the PA resources and/or facilities present in their school communities.

Although most of the girls were familiar with available resources and activities at their school, the activities available (e.g., swimming, football, track, and volleyball) were not always ones that the girls were interested in. Girls recognized physical education class as a source for activity, but many of them stated that they did not engage in regular physical education classes. Another challenge some girls faced when identifying ways to be physically active at school is that they attend schools where these resources are limited, unavailable, or underutilized (Table 1).

Girls also expressed concerns about their safety near school. In several cases for girls who attended school in an urban area, girls reported that school administrators prohibited students from traveling off-campus during school hours to nearby parks or fields, where danger may be present (Table 1).

### 3.3. Home Food Environment

Generally, an array of healthy and unhealthy foods was available in homes at any given time. Majority of the girls’ mothers repeatedly stated that they try to regularly provide healthy foods for their families with some families even imposing a “no junk food” rule, which usually did not last very long. However, parents felt that, when available, junk food should be given in moderation. Most girls usually did not initially agree with their mothers regarding restricting food but occasionally found merit in the unavailability of some unhealthy foods. There were several girls who indicated that they rebelled against the no junk food ban in the home and would circumvent the rule by purchasing their own food (Table 2).

Saving money was a major concern for most mothers, who adopted several shopping behaviors or systems for finding ways to stretch their budgets. Cost-cutting strategies included: shopping at multiple stores (i.e., farmers markets, Asian food stores, various grocery stores, and bulk food stores) for the best price, utilizing financial assistance (i.e., Supplemental Nutrition Assistance Program (SNAP) and Special Supplemental Nutrition Program for Women, Infant and Children (WIC)), couponing, and only purchasing sale items. Pressures to save food or reduce food spending can also affect food dynamics at home. Girls indicated that they were often asked to eat everything on their plates and save uneaten food for the next meal (Table 2).

Comments made during the focus group indicated that girls exercise a lot of power to persuade parents to purchase the foods that they want. Factors that influence a parents’ choice to honor their daughter’s requests include: deliberating with the other parent, the mood parent is in at the time, cost, nutritional value, rare requests, and whether the child’s request was planned in advance of the shopping list being made. In other instances, girls would purchase the foods they want with money they received from allowances, birthdays, or their own jobs (Table 2).

Girl’s excessive snacking during the day or night was a common concern among mothers and a frequent behavior among girls (Table 2). Another middle school mom shared that, because she did not set rules, her daughter would eat a calorie-dense snack shortly after eating dinner. The mom felt that her daughter was not still hungry but consumed the additional calories out of boredom or habit. Late night snacking appeared to be more prevalent among high school girls, who reported eating past dinnertime. This is a behavior that the mothers did not support.

In addition to excessive snacking, girls and mothers described the necessity to store food in various places (sometimes personal refrigerators) in the girl’s room because it may be forbidden food not usually allowed in the house, food the girls may have purchased themselves, foods that were purchased by parents specifically for the girls, or foods they want to prevent from being consumed by others living in the house.

### 3.4. School Food Environment

With respect to the school food environment, several factors were discussed. Girls reported a vast selection of meal options at school and in their immediate school environments. At school, girls chose between standard school lunches or snacks/meals from vending machines and/or school stores. Typical lunchroom choices included chicken nuggets, spaghetti, pizza, fries, burgers, sandwiches, fruits, salads, milk, and water, while usual vending machine or school store options included chips, pastries, candy, juice, ice cream, and coffee. In addition to healthy and unhealthy food options available in school, girls were also exposed to an assortment of off-campus dining/food options (i.e., fast food restaurant chains, healthy fast food restaurant chains, pizza chains, average-priced grocery stores, corner stores, coffee shops, specialty food stores, and drugstores) either in walking distance or a short bus or car trip ride away. In several instances, girls also said school administrators catered food from local food establishments or allowed restaurants to solicit to students through free food giveaways throughout the week.

Girls communicated mixed perceptions of the unhealthy food options available around their school communities. Within walking distance, high density food communities featured more fast food chains, unhealthy dine-in restaurants, discount food/retail stores, average-priced grocery stores, Chinese restaurants, malls with food courts, and corner stores. A few older girls shared that they frequented certain restaurants in their neighborhood because they received employee discounts. Equally, girls also commented on attending school (and/or living) in food areas with limited food options and availability. Types of food options were primarily gas stations and corner stores, although a few pizza/wing restaurants or Chinese/African restaurants were named (Table 2). In a few cases with girls who attended school in suburbs or downtown, no food options were identified within close proximity of the school.

### 3.5. Rules within the Home Environment

Daughters and mothers rarely mentioned any rules related to PA that were established within the home, except the inability to play outside due to safety concerns or only after their homework or chores were completed (Table 3).

There were more consistent food-related rules than any other household rule reported by girls and mothers, such as prohibiting food past a certain hour at night, prohibiting food consumed outside the kitchen or dining room table, mandatory family meals (“*If it’s dinner, we all have to eat up at the table together*.”), clean plate rule, prohibiting the use of excess salt, cleaning up after meals and snacks, prohibiting junk food before meals and breakfast, sitting down to eat rather than standing, exercising portion control, eating vegetables at dinner, snacking on healthy foods only, no eating while multitasking (i.e., doing homework or watching TV), and no shopping at convenience stores (i.e., gas stations and corner stores). Some of the rules that were harder to comply with were: eating past a certain hour, eating in designated rooms only, eating all the food on plate, and serving well-balanced meals. These rules were harder to comply with due to inconsistencies in parental behavior (Table 3). Middle school and high school girls also reported that snacks, drinks, and candy are allowed in bedrooms while meals are prohibited (Table 3).

In many households, TVs were commonly located in or near eating areas or directly in the kitchen, making eating in front of TVs effortless. A majority of the girls reported that their parents allowed them to watch TV while eating a snack or a meal. Multiple girls stated that family meals were eaten in front of the TV and planned around a particular TV program (Table 3). Although some girls reported eating full meals in front of the TV, they primarily consumed snacks. Popular snacks listed by girls were ramen noodles, cereal bars, sweets (i.e., candy, ice cream, and pastries), chips, popcorn, and sunflower seeds.

In many households, parents set rules that limited children’s, particularly younger children’s, access to watching TV only after completion of chores and/or homework or on weekends (Table 3).

To reduce younger girls’ TV viewing, some middle school mothers indicated that they removed TV from their children’s bedrooms, but not always from their own room (Table 3). Despite the removal of the bedroom TVs, most of the girls reported that they were not affected greatly. The older girls felt that they did not have enough time to watch TV due to school constraints, preference for the computer or phone, or the option to use a TV located in another room. The vast majority of high school girls reported having no rules related to TV consumption. Instead, girls stated that parents trusted the girls’ judgment and ability to self-monitor their behavior. High school girls did report watching TV and doing school work at the same time and staying up late to watch TV.

Enforcement of rules varied across households, but rules related to TV watching were more inconsistently enforced than other household rules. These inconsistencies were mainly related to agreement of the rule, enforcement, and to which family members the rule applies. One parent would agree that rules related to TV viewing should be in place, while the other parent did not feel the same. Both daughters and mothers reported that certain rules were made, but would not be enforced (Table 3). Lastly, there appeared to be a discrepancy between which family members are governed by certain TV rules. Some TV policies did not apply to every family member, such as husbands, parents, or older children.

## 4. Discussion

This qualitative study examined socio-cultural and environmental factors (i.e., related to availability and accessibility) that contribute to PA and health eating behaviors of African-American girls as reported by girls and their mothers. Major findings were girls are exposed to a wide range of PA and food resources—i.e., some girls reported lots of options and resources available, while others reported very few. Access to their PA environments is influenced by personal choice (i.e., desire, willingness, motivation), barriers (i.e., cultural, safety, school policy), and family or peer involvement. Personal choice to exercise brings up an important issue, that even with the most conducive environment, some girls may not engage in regular PA. The availability of PA resources alone is not enough to sustain PA, but targeted efforts need to be made to girls, parents, and school and community authority [25]. For this particular population, addressing motivating factors for PA and the influence of other social support such as parental or peer support on PA level, along with availability to PA opportunities, are viable strategies to increasing activity. 

Another important finding is the positive influence of social or family support on PA level; many participants described being active with their families and friends. Although the current study focused on a single sex, other work incorporating both male and female adolescents have reported peer and family support to be important facilitators of PA [26,27], which further corroborates the need for intervention strategies to incorporate activities that would be feasible for not only adolescents, but members of their social circles, such as a friends and family members.

Both daughters and mothers reported that rules related to eating (i.e., eating time, eating spaces, and eating while watching TV) were used to control the eating behaviors of families, though there appeared to be some discrepancy between the implementation of those rules in the household (i.e., rules do not apply to certain family members, rules are relaxed at specific times or occasions, or rules are not enforced). Additionally, there were rules preventing girls from being active, ranging from unfair or unequal opportunities to safety concerns. Safety was a major barrier reported among participants. This singular factor is particularly of importance, especially in adolescent girls’ homes, where parents may not have time to take their daughters to the gym or sport practice and do not feel they are safe to go on their own. African-American girls are more likely to have less PA equipment and resources within the home than other girls [28]. It is a common belief that parents may be more willing to enroll boys for sports activities rather than girls, which may be partly attributed to the general assumption that boys can protect or defend themselves better than girls [10]. Safety concerns should be explored in the context of home and school environment, particularly as African-American neighborhoods are more likely to be low income, densely populated and perceived to be unsafe. Larson et al. reported the perceived lack of safety is associated with a negative weight outcome [29].

There are various food environments that girls access at and around school (i.e., convenience stores and fast food restaurants), in and outside their immediate communities (i.e., neighborhood grocery stores and food courts at the mall), and at home (i.e., from the kitchen or stashed in their bedroom). In a sample of high school girls, Bauer et al. found that the African-American girls reported a moderate degree of availability of unhealthful foods, such as sugar sweetened beverages, salty snacks, and candy within the home (8.9 out of a possible 16 score) [28]. From the focus group discussions of the current study, most girls reported easy accessibility to similar unhealthy food choices both in the school and at home, which can play a central role in their choices (and challenges) to eat healthy. 

In the present study, mothers reported that economic reasons and convenience (i.e., closeness of stores, scheduling) dictate where they shop for household foods, but food requests from girls are granted by appealing to parents or peers or by purchasing food using personal money (allowances, gifted money, or work). It is well established that children’s “nagging power” is highly effective and influential on food purchasing decisions of parents [30]. Interviews with parents of children and young adolescents revealed that parents are repeatedly approached by their children to buy unintended, and often unhealthy, foods and drinks [31]. Although parents were not expecting to make the purchase and often felt that the food was unhealthy, 70% of the time, parents made the purchase anyway. “Nagging power” is a real phenomenon that can lead to the availability and accessibility of more high-calorie, low-nutrient foods. The participants from the current study reported similar nagging behavior, in addition to using their own money to purchase snack foods and drinks instead of eating school lunch. This, too, is normal adolescent behavior and has been reported in a recent study [32], with the consequence of making unhealthy foods available.

### Strength and Limitations

In the obesity prevention literature, little to no research has been conducted to investigate the environmental factors that influence middle and high school aged African-American girls’ and their mothers’ PA and healthy eating behaviors. However, research conducted with African-American girls ages 8–10 years old and their parents revealed similar findings with respect to girls having access to PA resources and family members acting as co-participants and facilitators of PA [33]. In a study conducted with an ethnically diverse group of mothers of children ages 2–5 years old [34], most African-American mothers reported accommodating child’s specific food requests and preparing foods that their child preferred, which was also a major theme of the current study. In a study by Berge et al. (2012), black and white families reported that accessibility to healthy eating and PA resources, family investment (i.e., rules related to sedentary behaviors), and parent modeling of health behaviors were important factors of eating healthily and being physically active [35]. The same themes consistent in the current research of children’s influence over parents’ food purchasing, children’s ability to prepare meals for themselves, and child food preferences were major points discussed in a focus group study with children ages 8–11 years old [36]. The current study represents one of few qualitative research studies to explore the dynamics of obesity-related behaviors among African-American girls and their mothers using a comprehensive socio-ecological model.

A limitation of the current study is that no physiological or survey measures were collected at the time of the focus groups. This information could have been useful in providing context to the knowledge, opinions, and beliefs reported by African-American girls and mothers. Furthermore, collecting these data could have developed a deeper understanding of subgroup differences, such as income, education, and current health behaviors. Factors such as these provide another layer of complexity to the environmental factors reported on during the discussion.

## 5. Conclusions

Developing a comprehensive understanding of the socio-ecological factors that influence the obesity-related behaviors of African-American female populations is urgently needed in public health research and practice if there is any hope to address the disparities between this group’s and other group’s health outcomes. Many of the results reported from this study reinforced what is known about facilitators and barriers to regular PA and healthy eating in adolescents, but provided additional evidence specific to African-American adolescent girls. The information collected from the focus groups can be used to better inform the development of obesity prevention and treatment studies targeting this population. The data collected in the current research hope to fill literature gaps by providing more contextual evidence for what is commonly known or assumed about obesity prevention and treatment for African-American female populations. Although many topics were addressed, there appears a need to further explore cultural beliefs underpinned by rules such as no eating of certain foods, no eating in the bedroom, and no watching TV at certain time. Further focus groups are needed to assist in intervening on home, school, and community environments that the current research has confirmed to be key to future intervention delivery systems. In future qualitative studies, attention should be placed on constructing nested sampling frames to control for demographic differences among African-American girls and women that may help to tailor interventions in a more responsible and effective way for these subpopulations.

## Figures and Tables

**Table 1:** Supporting quotes for factors in the home and school environments that influence physical activity.

**Home Physical Activity Environment**	
Unstructured physical activity	“*I actually like to bike around [NAME OF LAKE] a lot or [NAME OF ANOTHER LAKE] but I’ll just go walking by my house.*” - High school girl
Family members, friends, peers and pets with whom they can be active	“*We have a family Y, so it’s always cool if her friends come over…they can do that but they find clubs and stuff to go to workout*.” - Mom of high school girl on availability of friends with whom her daughter can be active
Structured sports within the community	“*It’s just a dance team that I’m on and we have to do different things before we even start dancing. So, like for the first thirty minutes —we ran around the park for thirty minutes to just warm-up and stuff like that*.” - High school girl
Perceived safety related to neighborhood crime and violence	“*To be honest with you I’d be kind of scared ‘cause I grew up right you know, I was raised here in the Cities and there was so much stuff going on. I’m like, do I want to sit outside with bees, mosquitoes and shooters?*” - High school girl
**School Physical Activity Environment**	
Available school facilities	“*[DAUGHTER’S NAME] is doing ballet and pointe…at her school. She can do ballet for PE, and she’s also taking a class after school on Wednesdays and Thursdays and that’s two hours on both of those days and an hour each day at her school so that’s what she’s doing*.” - Mom of high school girl
Limited, unavailable, or underutilized school facilities	*“I like going to like a black school* [i.e., a school where majority of the students categorized as black or African-American] *but I wish there were more sports because there’s no sports but, I mean there’s a lot of activities you can do in Phys Ed, so I just don’t get why we have like a big field outside, but we just don’t get to use it for track and stuff*.” - High school girl
Discouraged use of resources	“*A lot of people bike into school, yeah, and there are biking trails that go by the school… [SCHOOL ADMINSTRATORS] don’t really encourage walking by yourself considering [NAME OF PARK] is right over there and [PARK] is kind of sketchy*.” - High school girl

**Table 2:** Supporting quotes for factors in the home and school environments that influence food Choices.

**Home Food Environment**	
Availability of healthy and unhealthy foods	“*When my mom did it <banned junk food>… my brother and I kind of looked at each other like what is she doing? And she said no salt. That’s why it’s kind of hard for me to eat salt now.*” - High school girl “*My mom tried to tell me that I couldn’t eat candy. I just bought it at school. She was like, ‘No she doesn’t eat candy when I’m around.’ Okay, I eat candy all the time*.” - High school girl
Purchase own food	“*I work at a grocery store so I just basically buy all the foods that I want, but if I ask my mom to go grab something ‘cause I want it, she’ll do it*.” - High school girl
Concern of wasting food	“*My mom don’t like to waste that money or that food. You better clean that plate*.” - High school girl
Excessive snacking	“*I saw her go into the freezer to get them, but my back was turned. I did hear the microwave, but then I saw when she pulled out six of those <frozen taquitos>.*” - Mom of middle school girl
**School Food Environment**	
Meal selection	
Accessibility to fast food restaurants	“*There isn’t really any fast food by my house, but there’s a grocery store—one <grocery store> so that would be the closest or the gas station*.” - High school girl

**Table 3:** Quotes illustrating home rules that influence physical activity and food choices.

Physical activity rules	“*I can’t go outside like from Monday through Thursday so I’ve got to stay in the house and find something to do…because when you stay outside for too long you don’t think about your homework or nothing...you want to go outside more than you want to do your homework*…” - Middle school girl
Food-related rules	“*Mother says don’t eat in the living room, but my mom is one of the people who like, she’ll say it and then she’ll see you doing it and won’t say nothing about it.*” - Middle school girl “*Not food, like food that you serve at the dinner table. But like, candy and treats and fruit snacks.*” - Middle school girl
Eating while watching TV	“*We try not to eat food on the couch but then once there’s some program on the TV that we’re all watching and we’ve made dinner and all that, we all eat on the couch.*” - Middle school girl
TV rules	“*My daughter—my kids are older now, but when they were younger, the rules were you can’t watch TV until your school work is done and no video games until Friday night. We started that when they were early <young> so they were kind of used to that*.” - High school mom
TV in bedroom rules	“*I took the TVs <out of my children’s bedrooms> ‘cause I think last year it was a distraction to them. They come home, sit right in front of the TV, and do their homework…I [took] the TVs and put ‘em in the basement*.” - Mom of middle school girl“ *For us, my mom says we’re not allowed to watch TV but she doesn’t enforce it…we’ll be watching, <and she would say> “you know you’re not supposed to have the TV on” and we’ll just kind of look and keep doing our homework and watching*.” - Middle school girl
